# Treatable Hyperkinetic Movement Disorders Not to Be Missed

**DOI:** 10.3389/fneur.2021.659805

**Published:** 2021-12-01

**Authors:** Aurélie Méneret, Béatrice Garcin, Solène Frismand, Annie Lannuzel, Louise-Laure Mariani, Emmanuel Roze

**Affiliations:** ^1^Département de Neurologie, Hôpital Pitié-Salpêtrière, AP-HP, Paris, France; ^2^Sorbonne Université, Institut du Cerveau - Paris Brain Institute - ICM, Inserm, CNRS, Paris, France; ^3^Service de Neurologie, Hôpital Avicenne, APHP, Bobigny, France; ^4^Département de Neurologie, Hôpital universitaire de Nancy, Nancy, France; ^5^Département de Neurologie, Centre Hospitalier Universitaire de la Guadeloupe, Pointe-à-Pitre, France; ^6^Faculté de Médecine, Université Des Antilles, Pointe-à-Pitre, France; ^7^Centre D'investigation Clinique Antilles Guyane, Pointe-à-Pitre, France

**Keywords:** hyperkinetic, movement disorders, treatable, autoimmune, infectious, metabolic, genetic

## Abstract

Hyperkinetic movement disorders are characterized by the presence of abnormal involuntary movements, comprising most notably dystonia, chorea, myoclonus, and tremor. Possible causes are numerous, including autoimmune disorders, infections of the central nervous system, metabolic disturbances, genetic diseases, drug-related causes and functional disorders, making the diagnostic process difficult for clinicians. Some diagnoses may be delayed without serious consequences, but diagnosis delays may prove detrimental in treatable disorders, ranging from functional disabilities, as in dopa-responsive dystonia, to death, as in Whipple's disease. In this review, we focus on treatable disorders that may present with prominent hyperkinetic movement disorders.

Hyperkinetic movement disorders comprise dystonia, chorea, myoclonus, tics, stereotypies, and tremor, which can be isolated or associated with other neurological or non-neurological signs. Possible causes are numerous, and prioritization might prove challenging for clinicians. Whereas some diagnoses may be missed or delayed without serious consequences, diagnosis delays may prove detrimental in treatable disorders, which should, therefore, always be at the forefront of investigations (see Figures 1, 2 for an overview). Whereas metabolic, drug-induced and functional disorders are quite frequent, autoimmune, infectious and genetic causes are rare. In this review, we focus on disorders that may present with prominent hyperkinetic movement disorders, and thus be referred primarily to movement disorders specialists. We included disorders that could be fully cured or significantly improved with either pathogenic or symptomatic treatment.

**Figure 1:**
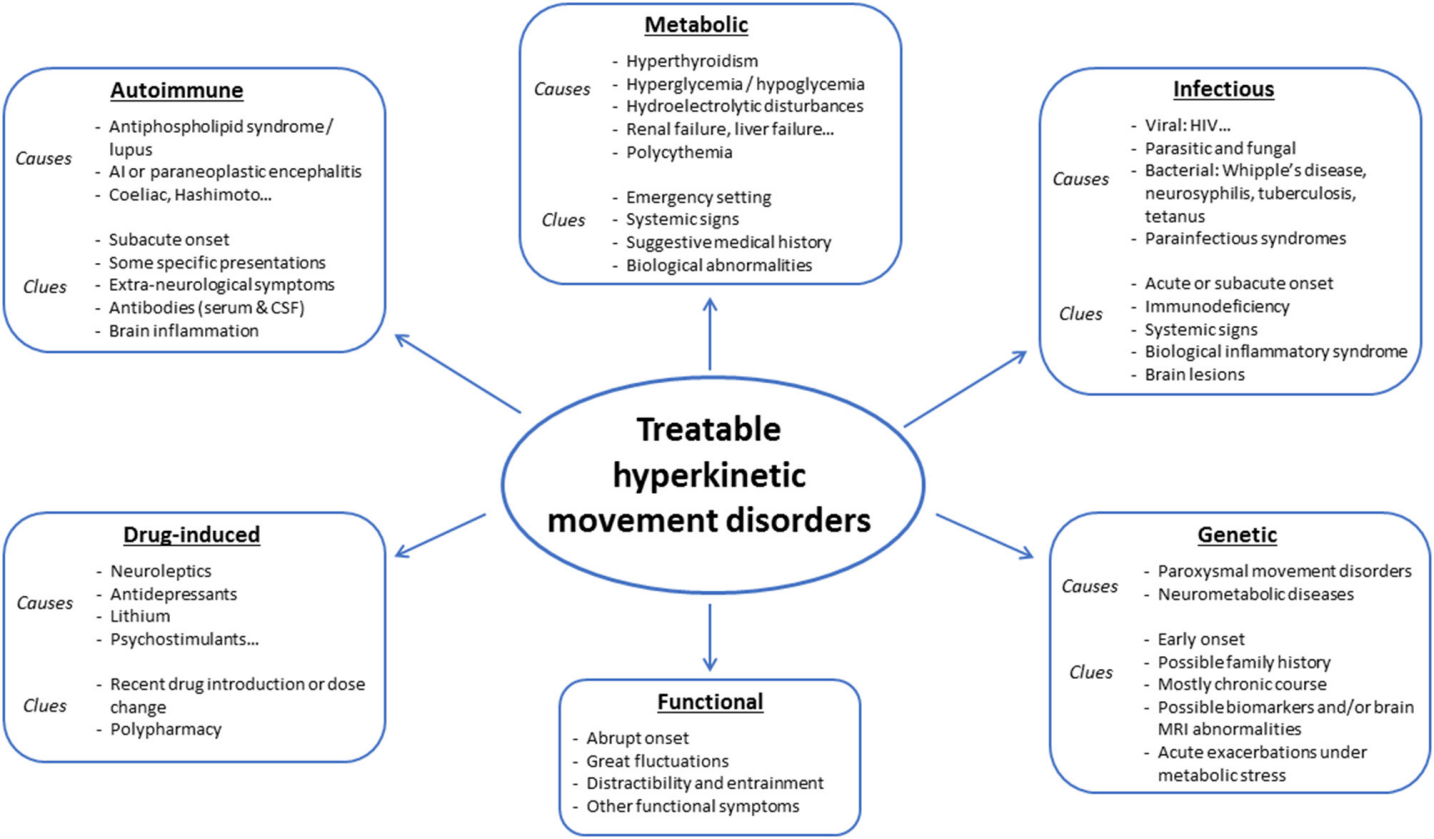
Main causes of treatable hyperkinetic movement disorders.

**Figure 2:**
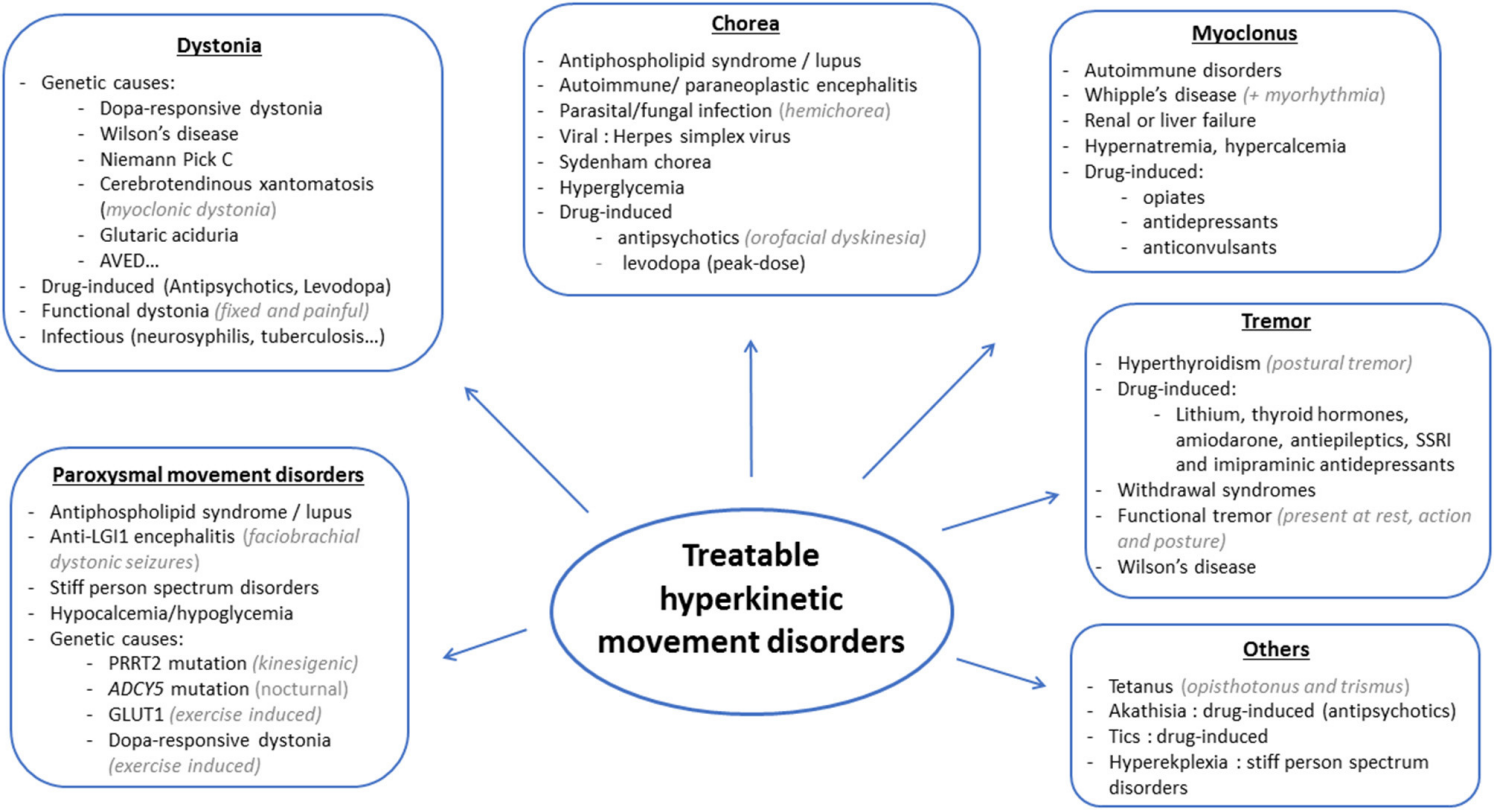
Main causes of treatable hyperkinetic movement disorders based on the phenomenology.

## Autoimmune Diseases

Hyperkinetic movement disorders are a common presenting feature of autoimmune disorders, often associated with other neurological and non-neurological symptoms. In addition, some presentations are very evocative of a specific cause, such as faciobrachial dystonic seizures in anti-LGI1 encephalitis. An autoimmune work-up and a brain MRI should therefore always be done in patients presenting with movement disorders of a subacute onset. The main causes and findings are summarized in Table 1.

**Table 1:** Autoimmune causes of hyperkinetic movement disorders.

**Cause**	**Movement disorders**	**Laboratory findings**	**Brain MRI findings**	**Treatment**
Antiphospholipid syndrome	Chorea +++ PNKD, myoclonus, dystonia, ataxia	Antiphospholipids (lupus anticoagulant, anti-cardiolipin, anti-β2-GP1)	Normal or ischemic strokes	Antithrombotic therapy
Lupus	Chorea +++ PNKD, myoclonus, dystonia, ataxia	Anti-DNA antibodies	Normal or non-specific abnormalities	Immunotherapy
Anti-NMDA encephalitis	Orofacial dyskinesia, Chorea, dystonia, myoclonus	Anti-NMDA antibodies	Possible T2 hyperintensities	Immunotherapy +/- cancer treatment
Anti-LGI1 encephalitis	Faciobrachial dystonic seizures	Anti-LGI1 antibodies	Possible T2 hyperintensities	Immunotherapy
Stiff person spectrum disorders	Fluctuating muscle rigidity, spasms, hyperekplexia, myoclonus	Anti-GAD antibodies +++ Anti-GlyR, anti-amphiphysin, anti-DPPX	Possible T2 hyperintensities	Immunotherapy
Anti-CV2/CRMP5, anti-D2R, anti-Hu encephalitis	Chorea	Anti-CV2/CRMP5, anti-D2R, anti-Hu antibodies	Possible basal ganglia T2 hyperintensities	Immunotherapy
Anti-IgLON5 disease	Chorea, myoclonus, ataxia	Anti-IgLON5 antibodies Polysomnography abnormalities Central hypoventilation	Normal or brain atrophy	Immunotherapy
Coeliac disease	Chorea, myoclonus, restless legs syndrome, dystonia, tremor, paroxysmal dyskinesia, ataxia	Anti-transglutaminase and/or anti-gliadin antibodies, Positive duodenal biopsy		Gluten-free diet
Hashimoto's encephalopathy	Myoclonus, dystonia, tremor, chorea	Anti-Tg and anti-TPO antibodies, Possible thyroiditis	Possible T2 hyperintensities	Immunotherapy

*PNKD, paroxysmal non-kinesigenic dyskinesia; Tg, thyroglobulin; TPO, thyroid peroxydase. ^+++^Most frequent*.

### Antiphospholipid Syndrome/Systemic Lupus Erythematosus

Movement disorders, particularly chorea, are well-known complications of antiphospholipid syndrome (APS) and systemic lupus erythematosus (SLE) (1, 2). Although these two disorders are often combined, movement disorders have been described in APS without SLE, and in rare cases of SLE without antiphospholipid antibodies (aPL) (2, 3). APS is usually defined by the occurrence of recurrent thrombosis and/or pregnancy complications in the presence of persistent aPL (lupus anticoagulant, anti-cardiolipin or anti-β2-glycoprotein-1, present on two occasions, at least 12 weeks apart) (1, 4). Movement disorders may be a consequence of ischemic strokes, but often occur without any evidence of ischemic damage. They are reported in <5% of patients with APS and SLE but may be the sole presenting manifestation (5, 6). In this setting, the most common movement disorder is chorea, which may be unilateral or generalized (often asymmetrical). Other movement disorders reported in APS and SLE include paroxysmal non-kinesigenic dyskinesia, myoclonus, dystonia, parkinsonism, and cerebellar ataxia (2, 7). Other causes, such as coincidental Parkinson's disease, must of course be excluded before confirming the diagnosis. Pathogenesis may involve both thrombotic and immunologic processes, as responses to either antithrombotic or immunosuppressive therapy have been reported. Other processes might be involved as well, since APS and SLE are causes of chorea gravidarum, which remits after delivery in most cases (8). Of note, the occurrence of thrombotic events is frequent over the disease course in patients with isolated chorea at the disease onset (9). Chorea and paroxysmal dyskinesia may abate significantly or even disappear with antithrombotic treatment alone, even without evidence of ischemic damage (2, 7, 9, 10). Therefore, antiplatelet or anticoagulant therapy is recommended in all patients, the latter preferred for patients with thrombotic APS (11). Dopamine antagonists may be used as well in patients with disabling hyperkinetic movements, particularly during the initial phase. Immunotherapy may be considered in non-responders and patients with additional autoimmune manifestations.

### Autoimmune or Paraneoplastic Encephalitis

Movement disorders of all kinds are a common feature of autoimmune and paraneoplastic encephalitis, either at the forefront of the phenomenology or as part of a larger phenotype (12, 13). The subacute onset of movement disorders is an important clue to the diagnosis, which should prompt the search for autoantibodies in the serum and the cerebrospinal fluid (CSF). Early and adequate diagnosis is key, as most patients respond well to immunotherapy and/or treatment of an underlying cancer, especially in cases of autoantibodies targeting cell-surface antigens, less so with antibodies against intracellular antigens (12).

Chorea and cerebellar ataxia may be seen in various types of autoimmune and paraneoplastic encephalitis, so that their acute or subacute occurrence should always prompt the search for autoantibodies targeting either cell-surface or intracellular antigens. Here, we will review a few examples where hyperkinetic movement disorders are either highly predictive or the main symptom of a particular disorder.

Anti-N-methyl-D-aspartate (NMDA) receptor encephalitis is the most frequent autoimmune encephalitis, particularly in young women (13). It is frequently associated with an ovarian teratoma. Diagnostic delay is frequent, as patients may present solely with psychiatric or cognitive symptoms at the onset. Various movement disorders are common, often with a very wide range of abnormal movements that are difficult to classify with standard definitions (3). Orofacial dyskinesias are very evocative of anti-NMDA encephalitis when the onset is subacute and unrelated to chronic neuroleptics exposure.

Anti-LGI1 encephalitis, also quite frequent, is characterized by a movement disorder that is almost pathognomonic, i.e., faciobrachial dystonic seizures. Rapid diagnosis and treatment are keys to prevent the development of cognitive impairment (14).

Another specific clinical presentation not to be missed concerns Stiff person spectrum disorders (SPSD), which include stiff person syndrome, stiff limb syndrome, stiff person plus (with additional symptoms), and progressive encephalomyelitis with rigidity and myoclonus (PERM) (13, 15). Clinically, they are characterized by fluctuating muscle rigidity, spasms, and hyperekplexia, inducing frequent falls, and may be fatal in the case of PERM. In addition, anxiety and specific phobias such as pseudoagoraphobia, i.e., gait-specific phobia, are frequent features of stiff-limb and stiff-person syndrome (16, 17). Due to the associated psychiatric symptoms, the fluctuating nature of the rigidity and sometimes unexplained falls, SPSD are often misdiagnosed as functional disorders. Associated antibodies are mainly anti-GAD, followed by Glycine receptors antibodies, anti-amphiphysin and DPPX, the latter further characterized by gastrointestinal symptoms with severe weight loss (18).

Chorea may be seen in patients with anti-CV2/CRMP5, anti-Dopamine D2 receptors (D2R), or anti-Hu (19). It is usually combined with other neurological signs and MRI FLAIR hyperintensities, but may also be isolated (10% of CV2/CRMP5 cases) and with normal MRI or with a caudate atrophy suggestive of Huntington's disease.

Finally, wrongful diagnoses of progressive supranuclear palsy or multiple system atrophy may be made in cases of anti-IgLON5 disease, characterized by various combinations of hypo- and hyperkinetic movement disorders, sleep abnormalities, cognitive impairment, bulbar signs, and respiratory dysfunction (12, 20). A subacute onset and rapid worsening are red flags that should prompt the search for anti-IgLON5 antibodies. First believed to be mainly a neurodegenerative disorder of poor prognosis, anti-IgLON5 disease more and more appears to involve autoimmune processes, with recent reports of good responses to aggressive and sustained immunotherapy, advocating for the importance of early diagnosis and treatment in this disorder (21).

### Other Autoimmune Diseases

Coeliac disease, or gluten-sensitive enteropathy, is mostly characterized by gastrointestinal symptoms, but may be accompanied by neurological signs (22, 23). Cerebellar ataxia is the most common neurological presentation, but varied hyperkinetic movement disorders such as chorea, myoclonus, restless legs syndrome, dystonia, tremor and paroxysmal dyskinesia may be seen (24). In particular, cortical myoclonus, characterized by prominent stimulus sensitivity, is quite frequently seen in Coeliac disease (CD) (25). Diagnosis is suggested by the positivity of anti-transglutaminase and/or anti-gliadin antibodies and may be confirmed by duodenal biopsy. Treatment with gluten-free diet has been reported to improve or even suppress movement disorders in some patients but is often ineffective on myoclonus (24–26).

Hashimoto's encephalopathy may also present with ataxia and/or movement disorders, namely myoclonus, dystonia, tremor or chorea, with or without clinical signs of encephalopathy (3, 27). Diagnosis should therefore be suspected in any patient with unexplained movement disorders, and anti-thyroglobulin (Tg) and anti-thyroid peroxidase (TPO) dosed in serum and CSF even when thyroid hormone levels are normal (27). Caution should, however, be applied before confirming the diagnosis, as these antibodies are quite frequently found in the general population. Treatment involves corticosteroids and intravenous immunoglobulins.

Although movement disorders are not common features of Sjögren syndrome, subacute chorea has been reported in rare cases, with treatment involving corticosteroids or immunosuppressive drugs (28, 29). Hyperkinetic movement disorders may also be seen in neuro-Behcet's disease (30, 31).

## Infectious Diseases

Many infections of the central nervous system (CNS) have an affinity for the basal ganglia and can result in a variety of movement disorders. The movement disorders can be (i) the consequence of infectious space-occupying lesions located in the basal ganglia or other regions implicated in motor control, (ii) the manifestation of an acute infectious encephalitis, or (iii) the expression of a delayed immune-mediated process related to the infection. Infection may cause up to 20% of all secondary movement disorders (32).

This section will focus on treatable infection-related hyperkinetic movements that should be diagnosed as early as possible for optimal recovery. Treatments (summarized in Table 2) may comprise antimicrobial agents, which are the cornerstone of the treatment, symptomatic treatments during the acute phase, and immuno-active treatments in patients with delayed immune-mediated movement disorders.

**Table 2:** Antimicrobial management and immunoactive treatment of infectious and parainfectious hyperkinetic movement disorders.

**Disease**	**Diagnosis**	**Treatment**
HIV	Serology, P24 antigen RT-PCR in serum and CSF	Highly active combination antiretroviral therapy with high penetrance in the central nervous system
Toxoplasmosis	Serology and PCR in serum and CSF	Acute therapy: pyrimethamine 200 mg PO 1 time, followed by weight-based therapy (at least 6 weeks)^§^ Second- line therapy (intolerance): atovaquone PO 1,500 mg bid or clindamycine 30 mg/kg daily (tid or qid, max 2,400 mg)
Cryptococcosis	Cryptococcal antigen (CrAg) detection and culture in serum and CSF India ink staining of CSF	Induction therapy (at least 2 weeks): amphotericin B 3–4 mg/kg IV daily + flucytosine 25 mg/kg PO qid Consolidation therapy (at least 8 weeks): fluconazole 400 mg PO (or IV) daily Maintenance therapy (at least 12 months): fluconazole 200 mg PO daily
Histoplasmosis	Serologic tests and detection of histoplasma antigen in blood, urine and CSF	Induction therapy (4–6 weeks): liposomal amphotericin B IV 3 mg/kg/day once a day. Maintenance therapy: itraconazole 200 mg PO bid or tid for ≥12 months and until resolution of abnormal CSF findings followed when necessary by itraconazole 200 mg PO daily; therapeutic drug monitoring (10–14 days after starting, once monthly for follow-up) Second- line therapy (digestive intolerance or itroconazole level <1 mcg/mL): voriconazole, posaconazole
Cysticercosis	Serology in serum and CSF, PCR in CSF Limb X-Ray	Albendazole 15–30^¶^ mg/kg/day + praziquantel 50 mg/kg per day (15 days) + dexamethasone 0·1 mg/kg/day^*^^¬^ given 1–2 days before therapy
Whipple's disease	PCR in CSF, serum, saliva, feces PAS positivity on small bowel biopsy	Doxycycline 200 mg/day and hydroxychloroquine 600 mg/day for 12 months, followed by lifetime treatment with Doxycycline 200 mg/day
Syphilis	Troponemal (TPHA) and non treponemal (VDRL) tests in serum and CSF HIV serology, Brain MRI	First-line: benzyl penicillin 18–24 million units IV daily (10–14 day) Second-line (If hospitalization and IV benzyl penicillin is impossible): ∙ Ceftriaxone 1–2 g IV daily (10–14 days) ∙ Procaine penicillin 1.2–2.4 million units IM daily and probenecid 500 mg qid (both 10–14 days) Penicillin allergy: desensitization to penicillin followed by the first-line regimen Third-line: vibramycine 100 mg PO tid (10–14 day)
Tuberculosis	Chest X ray/CT scan AFB smear and culture (sputum, BLA X 3, CSF) PCR in CSF, Brain MRI, HIV serology	Intensive phase (2 months): isoniazid, rifampicin, pyrazinamide (in case of full susceptible M. tuberculosis); + ethambutol (when susceptibility non tested or resistance)^$^ + dexamethasone ^*^^$$^ Continuation treatment (7–10 months): isoniazid + rifampicin (unchanged daily dosage) multiresistance: moxifloxacine, linezolide, amikacin
Tetanus	Throat swab for GAS rapid test and/or culture (patient and contacts) ASO, ADB repeat after 2–6 weeks	Metronidazole 500 mg, IV, tid or penicillin G, 100,000–200,000 IU/kg/day Tetanus antitoxin: 500–3,000 IU, once IM Vaccination
HSV induced AE	HSV RT-PCR in CSF Anti-neuronal antibodies in serum and CSF	Intravenous aciclovir 10 mg/kg q8 h discontinued when HSV PCR negative in CSF on 2 occasions at least 24–48 hrs apart or when anti neuronal antibodies are positive in CSF First-line immunotherapy: corticosteroids, IVIG or plasmapheresis Second-line immunotherapy: rituximab, cyclophosphamide Maintenance therapy: oral corticosteroids, monthly IVIG, azathioprine, mycophenolate mofetil
Sydenham's Chorea (SC) (PANDAS)	Throat swab for GAS rapid test and culture (patient and contacts) ASO, ADB; repeat in 2–6 weeks	Penicillin V PO (10 days): children 250 mg/dose bid or tid; adolescents or adults 500 mg/dose bid or Amoxicillin PO 10 days 50 mg/kg once daily, maximum 1 g Penicillin allergy: clindamycin PO (10 days) 7 mg/kg/dose tid or clarithromycin PO (10 days) 7.5 mg/kg/dose bid or azithromycin PO (5 days) 12 mg/kg once or any other macrolide Chroea paralytica: prednisone (2 mg/kg/day) or methylprednisolone (25 mg/kg/day) for 3–4 weeks SC refractory to antibiotic therapy: IVIG or plasmapheresis Antibiotic prophylaxis: benzathine penicillin G IM once monthly 600 000–1.2 M U^£^ for up to 5 years
OMAS	Wide infectious assessment	Antimicrobial therapy when appropriate Immunotherapy: corticosteroids, IVIG or both

*HIV, human Immunodeficiency Virus; CSF, cerebrospinal fluid; TPHA, pallidum hemagglutination test; VDRL, Venereal Disease Research Laboratory test; AFB, acid-fast bacilli; PCR, polymerase chain reaction; RT-PCR, real-time polymerase chain reaction; HSV Herpes Simplex Virus; HSV AE, HSV-induced autoimmune encephalitis; PANDAS, Pediatric Autoimmune Neuropsychiatric Disorders Associated with Streptococcal Infections; SC, Sydenham's Chorea; GAS, group A beta-hemolytic streptococcus; ASO, antistreptolysin-O; ADB, anti-DNase B antibodies; BAL brochioalveolar lavage; OMAS, Opsoclonus-myoclonus-ataxia syndrome; IM, intramuscular; IV, intravenous; IVIG, intravenous immunoglobulin; PO, oral administration/per oral; bid two times daily; tid three times daily; qid four times daily, q (n) h = every “n” h*.

§ *If <60 kg, pyrimethamine 50 mg PO once daily + sulfadiazine 1,000 mg PO q6 h daily; If ≥60 kg, pyrimethamine 75 mg PO once daily + sulfadiazine 1,500 mg PO q6 h daily; + folinic acid 10–25 mg PO daily*.

¶ *Albendazole: 15 mg/kg/day for intra-parenchymal cysts; 30 mg/kg/day for subarachnoid and ventricular cysts*.

* *To avoid immune response due to pathogen lysis*.

¬ *Dexamethasone up to 16 mg/kg when intracranial hypertension, for 1–2 weeks or more, slow taper*.

$ *Isoniazid 5 mg/kg/day per kg; rifampicin 10 mg/kg/day; ethambutol 20 mg/kg/j kg; pyrazinamide 20–30 mg/kg/j*.

$$ *Dexamethasone IV 4 weeks (0·4 mg/kg per day for week 1, 0·3 mg/kg per day week 2, 0·2 mg/kg per day week 3, 0·1 mg/kg per day week 4) followed by 4 mg total of oral drug, reduced each week by 1 mg until zero was reached*.

£ *≤ 27 kg (60 lb): 600,000 U; >27 kg (60 lb): 1.2 M U; for up to 5 years in SC*.

### Hyperkinetic Movement Disorders Due to Viral Infection of the Basal Ganglia

Several neuroinvasive viruses with an affinity for basal ganglia can induce hyperkinetic movement disorders (33). In the absence of specific antiviral therapy, patients with virus-induced hyperkinetic movements receive symptomatic treatments and immunomodulation when appropriate. Direct brain invasion by HIV and the subsequent activation of the local immune response can result in a wide range of movement disorders, including chorea, tremor, myoclonus, dystonia, and paroxysmal dyskinesia (34–38). Typically, chorea or tremor are observed in patients with cognitive deterioration in the context of HIV encephalopathy or AIDS-dementia complex (35, 39, 40). Similar movement disorders can also be observed in patients with HIV CNS viral escape, who have an independent replication of HIV RNA in the central nervous system (despite a well-controlled plasma HIV infection), due to selective resistance to the treatment of the virus present in the CSF compartment (34, 41). Initiation or optimization of highly active antiretroviral therapies combinations, favoring drugs with good CNS penetration, is usually an effective treatment of movement disorders in this setting (34, 35, 41–44).

### Hyperkinetic Movement Disorders Due to Parasitic and Fungal Infections

Parasitic infections located in the basal ganglia and thalamus may induce contralateral limb ballism, choreoathetosis, and dystonia. Hemichorea–hemiballismus is the most frequent movement disorder in patients with cerebral toxoplasmosis, which is mainly observed in immunocompromised patients (33, 45). It remains rare, occurring in <10% of cerebral toxoplasmosis cases (46). Cerebral fungal infection should be considered in immunodeficient patients presenting with abnormal hyperkinetic movements. Hemichorea-hemiballismus and choreoathetosis have been reported to be associated with cryptococcosis [cryptococcal granuloma located in the subthalamic nucleus (47) or cryptococcal meningitis (48, 49)], or cerebral histoplasmosis (50) in HIV infected patients. Cases of hemidystonia or chorea were described in association with cysticercosis cysts or calcifications in the thalamus or putamen. Cysticercosis lesions are more often located in the cortex, where they may be responsible for postural and kinetic tremor of the upper limb, associated with epilepsy in 1/3 of cases (51). Of note, cortical lesions of cysticercosis can result in epilepsia partialis continuum, which can be misidentified as a hyperkinetic movement disorder. Anthelmintic therapy often results in full recovery of patients with movement disorders secondary to cysticercosis (52).

### Hyperkinetic Movement Disorders Due to Bacterial Infections

#### Whipple's Disease

Whipple's disease is a rare infectious disease (with an incidence of <1 per 1,000,000 a year) due to the bacterium *Tropheryma whipplei*, with CNS involvement in roughly a third of cases. Movement disorders are a common feature of Whipple's disease (WD), accounting for half of the cases with neurological symptoms (53). Other common neurological manifestations include cognitive alterations and vertical supranuclear gaze palsy. Most patients with neurological involvement also have systemic involvement (classically gastro-intestinal symptoms, arthralgia, and weight loss), but 5% of cases present with isolated neurological signs (53, 54). The most well-known, and virtually pathognomonic, movement disorders in WD are oculomasticatory myorhythmia (OMM) and oculofacioskeletal myorhythmia (OFSM). They are characterized by pendular oscillations of the eyes and concurrent masticatory or facioskeletal rhythmic and slow (1–4 Hz) contractions (55). However, OMM and OSFM are relatively rare in WD, occurring in <20% of cases with neurological involvement (53). Myoclonus and myorhythmia, as a whole, are the most common movement disorder in WD, followed by ataxia and Parkinsonism. Other movement disorders such as tremor, dystonia, and chorea are, however, rarely described (53).

Timely diagnosis and antibiotic treatment are crucial in this otherwise fatal disease. Diagnosis is based on PCR in various tissues and periodic acid-Schiff (PAS) positivity on small bowel biopsy. Optimal treatment type and duration are still debated, but most recent publications argue for a combination of doxycycline and hydroxychloroquine for 12 months rather than the historically favored sulfamethoxazole/trimethoprim regimen (53, 54, 56). Due to a high risk of relapse, some authors also suggest lifelong doxycycline treatment (56).

#### Neurosyphilis

Hyperkinetic movement disorders can be observed during late stage neurosyphilis, mainly due to lesions of the basal ganglia, midbrain, or cerebellum (57). Movement disorders result from ischemic lesions during the meningovascular phase, and from direct invasion and local inflammation during the parenchymal phase (57). They include chorea, myoclonus with cerebellar ataxia, orofacial and/or laryngeal dystonia (58–60). The diagnosis is based on the positivity of antigenic tests in the serum and/or the CSF (61). Early diagnosis and treatment are critical, as neurosyphilis can be a completely curable condition (61).

#### Others

Movement disorders may occur during tuberculous meningitis resulting from brain lesions with a frequency ranging from 0.5 to 18.6% (62). They can manifest as dystonia, chorea, ballism, myoclonus, or tremor (postural, kinetic, rarely at rest). Possible mechanisms comprise deep infarcts, hematoma, cerebral vasculitis, deep tuberculomas, arachnoiditis, hydrocephalus, or brain edema (63).

Tetanus, caused by toxins produced by the bacterium *Clostridium tetani*, is characterized by muscle spasms and autonomic nervous system dysfunction (64). Tetanus spasms can be induced by auditory, tactile, or visual stimuli. They are either generalized or localized to a limb or the head and neck. Trismus and dysphagia, pharyngeal and laryngeal spasms are typical early features of tetanus. In generalized tetanus, muscle stiffness and painful spasms predominating on the extensors can cause characteristic opisthotonus. Tetanus is a vaccine-preventable disease that still commonly occurs in many low-income countries. Although it is less frequent in high-income countries, 499 tetanus cases were reported in Japan between 2010 and 2016 (65), likely due to low immunity in old individuals. Mortality rate is almost 100% if left untreated. Acute treatment of tetanus is based on antibiotic eradication of Clostridium tetani, antitoxin, vaccination, and symptomatic treatment of rigidity and spasms (66).

### Parainfectious Hyperkinetic Syndromes

Chorea or ballism can occur after herpes simplex encephalitis (HSE) in about 25% of the patients (particularly in young children), usually 2–8 weeks after the initial HSE manifestations, with no specific imaging features (67–69). These patients have an HSV meningoencephalitis-induced autoimmune encephalitis, often with anti-NMDA antibodies. The pathogenic role of the anti-NMDA antibodies found in these patients and of other neuronal surface antibodies remains to be determined (67–69). The possible role of the resumption of viral replication (observed in some of these patients), although less likely, is still debated. Immune-active treatment is recommended in these patients (67–69).

Sydenham chorea is a hyperkinetic parainfectious syndrome associated with the group A beta-hemolytic streptococcus (GAS) infection. Involuntary movements generally involve the face and extremities. Hemichorea is observed in about 20% of cases. In “chorea paralytica,” hypotonia is severe and generalized, with absent or rare hyperkinetic movements (70). Psychiatric symptoms are frequent in Sydenham chorea and include emotional instability, irritability, anxiety, and behavioral changes. It is hypothesized that infection-triggered immune dysregulation may lead to antineuronal antibodies directed against specific targets in the brain (including neuronal lysoganglioside GM1, D1 and D2 dopamine receptors, and calcium calmodulin-dependent protein kinase II), eventually resulting in chorea and other neuropsychiatric symptoms (71). The diagnosis of Sydenham chorea is based on history and clinical findings supported by the detection of GAS by throat swab and/or the increase of either antistreptolysin-O (ASO) or anti-DNase B antibodies titers (72). Acute treatment is based on antimicrobial therapy active against acute GAS infection (72). In most cases, an improvement of symptoms is obtained within days or weeks (70, 73). Involuntary movements or behavioral disorders sometimes require additional symptomatic treatments. Long-term streptococcal prophylaxis is indicated in Sydenham chorea with multiple GAS-associated exacerbations (70, 74). Immune-active treatment options such as steroids, plasmapheresis, or intravenous immunoglobulins may be required in severe or refractory cases (70, 75).

PANDAS (Pediatric Autoimmune Neuropsychiatric Disorders Associated with Streptococcal infections) is a controversial disorder, mainly observed in males (74% of cases) and defined by the unusually abrupt onset of obsessive-compulsive symptoms and/or tics, which can be associated with additional psychiatric manifestations (73, 76). In the majority of cases, the first tics are motor and ocular (73). Basically, the pathophysiology, biological diagnosis markers, and treatment of PANDAS are similar to those of Sydenham chorea. One important issue is the frequency of positive throat cultures in the general population of children in the age group of interest that can confound the diagnosis of PANDAS. Other infectious agents than streptococcus might be involved in PANDAS (77). In particular, *mycoplasma pneumonia* and Varicelle-Zona virus can be considered as potential triggering factors for PANDAS, when GAS detection is negative (73).

Opsoclonus-myoclonus-ataxia syndrome is an immune-mediated movement disorder characterized by a variable association of opsoclonus, myoclonus, ataxia, behavioral, and sleep disturbances. It is mostly parainfectious or paraneoplastic (78, 79). Opsoclonus-myoclonus-ataxia syndrome (OMAS) has been reported in association with various infections including HIV, hepatitis C, Cytomegalovirus, human herpesvirus 6, adenovirus, enterovirus, rotavirus, influenza virus, dengue virus, West Nile virus, *Mycoplasma pneumoniae*, leptospirosis, psittacosis, *Salmonella enterica*, neuroborreliosis, and *Rickettsia conorii* (78, 80, 81). More recently it has also been associated with COVID-19 (82). HIV-related OMAS has been observed in recent infection with HIV, immune reconstitution, when another infection is associated (83), or in patients with CSF virus escape (84). In addition to the antimicrobial therapy (when appropriate), OMAS should be treated *per se* with corticosteroids and/or IVIG alone or a combination thereof (85).

## Acute Metabolic Disorders

Movement disorders may be observed in systemic metabolic disorders, as the presenting feature, later on within the disease course, or as a consequence of a corrective treatment. Other neurological manifestations may help the clinician to make the diagnosis, such as impaired consciousness, headaches, and seizures. A metabolic origin can also be suspected when movement disorders appear in emergency settings or in intensive care units. Treatment of the movement disorders in these cases mostly relies on the correction of the underlying metabolic disorder. This section will describe the main causes of treatable metabolic abnormalities inducing hyperkinetic movement disorders, which are outlined based on phenomenology in Table 3.

**Table 3:** Main treatable metabolic hyperkinetic movement disorders.

**Hyperkinetic movement disorder**	**Metabolic disorders**		**Diagnosis work-up**
	**Common**	**Uncommon**	
Tremor	Hyperthyroidism Chronic liver failure Chronic renal failure	Hypomagnesemia Hypercalcemia Vitamin B12 deficiency	TSH Hepatic check Renal check Magnesium, Calcium levels Vitamin B12, homocysteinemia Brain MRI
Chorea	Hyperglycemia	Polycythemia Hepatic failure Hypernatremia Hyperthyroidism Hypocalcemia Hypomagnesemia Vitamin B12 deficiency	Glycemia Hemoglobin level Hepatic check Sodium, Magnesium, Calcium levels Vitamin B12, homocysteinemia TSH Brain MRI
Myoclonus (positive and negative)	Chronic liver failure Chronic renal failure Hypernatremia Hypercalcemia	Severe hypophosphatemia Hypomagnesemia Metabolic alkalosis Vitamin B12 deficiency	Hepatic check Renal check Sodium, Calcium, Phosphorus, Magnesium levels Vitamin B12, homocysteinemia Blood gas
Paroxysmal disorders	Hypocalcemia Hypoglycemia Dysthyroidism		Calcium, Phosphorus levels, parathyroid hormone Glycemia TSH

### Liver Disease

Positive or negative myoclonus (asterixis) of acute or subacute onset is frequently found in hepatic encephalopathy (86). Their course parallels the evolution of the confusional state.

Other than parkinsonism, intentional and postural tremor is the most frequent movement disorder observed in chronic acquired hepatocerebral degeneration (CAHD). Two types of CAHD are described: a familial Wilsonian form and a non-familial form, which can be caused by several diseases. They comprise alcoholic cirrhosis, primary or secondary biliary cirrhosis, chronic hepatitis, portal-systemic anastomotic shunt, and hemochromatosis (87). MRI abnormalities consist in T1 hyperintensities mostly located in the internal pallidum, but also in the putamen. They are reversible after treatment of the hepatic disease. Pathogenic mechanisms may comprise: an accumulation of toxic substances such as manganese or ammonia, altered neurotransmission, neuroinflammation, and increased oxidative and nitrosative stress (88). Chorea may also be a manifestation of CAHD, typically with prominent orofacial, buccal, and lingual involvement, but may also be generalized. Choreic movements mostly appear to be independent from the hepatic encephalopathy episodes. Hepatic transplantation is, therefore, the only effective treatment in most cases, but ammonia-reducing therapy such as lactulose may improve movement disorders in some patients (89).

### Renal Disease

Patients with uremic encephalopathy may present with stimulus-sensitive myoclonus, asterixis, or both (86), which improve after dialysis or renal transplantation. Dialysis encephalopathy can also lead to myoclonus, along with dysarthria and seizures, caused by aluminum toxicity or rapid uremia reduction. Treatment is based on slow correction of uremia and avoidance of aluminum-containing dialytic fluids. Transient osmotic imbalance during dialysis can be associated with T2 hyperintense lesions of the basal ganglia (90). Various drugs may also induce myoclonus in the context of renal insufficiency. They include acyclovir, ciprofloxacine, dobutamine, cephalosporins, and gabapentin.

### Blood Sugar and Electrolyte Disturbances

Diabetic striatopathy is defined as a hyperglycemic condition associated with either one or both of: (1) chorea/ballism; (2) striatal hyperdensity on CT or hyperintensity on T1-weighted MRI of the basal ganglia (30). Hemichorea or hemiballism typically develops following severe non-ketotic hyperglycaemia in the context of long-standing poor control of type 2 diabetes mellitus (exceptionally type 1). Ketosis is not associated in most cases (91). It is more common in females. Most patients have hemichorea, but bilateral involvement is found in 10% of patients. Although choreic movements often dissipate within hours of hyperglycaemia correction, hemiballism may persist for over 3 months in 20% of patients. Delayed onset after achieving normoglycaemia, persistent severe movements or late recurrences are rarely observed. There may be T1-weighted MRI hyperintensities or hyperdensity on CT in the contralateral basal ganglia (putamen > caudate nucleus > globus pallidus) (91). Diabetic striatopathy (DS) differs from hypertensive hemorrhage by the absence of mass effect and the sparing of the internal capsule. Management involves treating the underlying cause, while protecting the limbs from injury. If severe or prolonged, symptoms can be alleviated by antipsychotics, dopamine-depleting agents, gamma aminobutyric acid (GABA)-receptor agonists, and selective serotonin reuptake inhibitors. Deep-brain stimulation of the globus pallidus or thalamus may be considered in refractory cases (92, 93). There are some evidence of hemorrhage as a possible pathogenic factor (91).

Hypoglycemia (mainly due to an insulinoma) and hypocalcemia are among the main causes of paroxysmal hyperkinetic movements due to systemic metabolic disorders. Diagnosis of paroxysmal disorders due to an insulinoma can be difficult, often resulting in a diagnosis delay (94, 95). The characteristics of the paroxysmal episodes are the following: (i) episodes can occur after prolonged exercise or during the second part of the night or early morning, (ii) paroxysmal symptoms increase and decrease gradually within an episode, (iii) mixed hyperkinetic movement disorders that include dyskinesia, dystonia, tremor, and myoclonus can vary from one day to another, and may last minutes to hours, (iv) movement disorders are often combined with behavioral disturbances, (v) drowsiness/dreaminess and dysautonomic manifestations can also be present. It is noteworthy that neuroglycopenic paroxysmal movement disorders may occasionally combine genuine abnormal involuntary movements and awkward (voluntary) movements reflecting behavioral disturbances. Glycemia is usually very low (0.2–0.4 g/L) during the episodes. Treatment mostly consists in the resection of the insulinoma, resulting in complete resolution of the paroxysmal episodes.

Hypocalcemia-related paroxysmal hyperkinetic movements often have a kinesigenic trigger and are typically characterized by asymmetric or unilateral dystonia, with involvement of the face (96, 97), or carpopedal spasms that can be mistaken for paroxysmal dystonia (98). Symptoms of hypocalcemia are highly sensitive to vitamin D and calcium replacement therapy.

Several neurologic abnormalities have been reported with hypomagnesemia or hypercalcemia, including tremor (99–101). In hypomagnesemia, tremor results from neuromuscular hyperexcitability, and may occasionally be associated with other disorders comprising opsoclonus, ataxia, chorea, and downbeat nystagmus (102). Chorea may also rarely be seen in hypernatremia and hypocalcemia (101).

### Polycythemia

Generalized chorea, hemichorea, or selective orofaciolingual chorea can be associated with primary or secondary polycythemia (103), either as an initial manifestation or as a sign of worsening. It is important to look for a *JAK2* mutation. Of note, chorea may occur in the absence of polycythemia in this disorder (104). The pathogenesis of chorea associated with polycythemia remains elusive, possibly involving dysregulation of neurotransmitter release, receptor sensitivity, and cellular metabolism. Chorea is often responsive to neuroleptics or dopamine depletors, along with serial phlebotomy and chemotherapy to treat the underlying disease process (105).

### Others

Postural and action tremor of the upper limbs can be observed in hyperthyroidism, isolated or associated with systemic features, such as tachycardia, diarrhea, and sweating. Of note, the presence of hyperthyroidism in Parkinson's disease usually precludes tremor control until it is identified and treated (106). More generally, hyperthyroidism can result in a wide range of movement disorders including chorea, ballism, athethosis, myoclonus, and dystonia, possibly because thyroid hormones interfere with dopaminergic pathways (107, 108). Chorea induced by thyroid hormones replacement has also been occasionally described (109). Treatment relies on the correction of the hyperthyroidism. Beta-blockers can also be used while waiting for the restoration of a normal thyroid function. In addition, both hypo- and hyperthyroidism can rarely manifest as paroxysmal dyskinesia that resolve with treatment of the thyroid disorder (110, 111).

Vitamin B12 deficiency is a rare cause of chorea, isolated or associated with more common manifestations of this disorder, such as combined degeneration of the spinal cord (112–114). Tremor and myoclonus have also been reported. Laboratory results show low Vitamin B12 levels and elevated homocysteinemia. Treatment involves oral or parenteral Vitamin B12 supplementation, depending on the underlying cause.

## Drug-Induced Hyperkinetic Movement Disorders

Drug-induced movement disorders represent about 20% of all movement disorders in adults over 50 (115), potentially avoidable or treatable, and about 3% of acute hyperkinetic movement disorders in children (116). Temporal and/or dose-response relationships between drug introduction and movement occurrence are highly suggestive of drug-induced movement disorders, in the absence of another known cause. This section will describe the main drugs responsible for various abnormal movements. The list of drug-induced phenomenology is outlined for the reader in Table 4.

**Table 4:** Drug-induced hyperkinetic movement disorders.

**Drug-induced movement disorders**	**Drugs involved**	**Comments**
Non-parkinsonian tremors	- Lithium, thyroid hormone replacement, amiodarone, beta adrenergic agonists, antiepileptics (valproate, carbamazepine, phenytoin), SSRI and imipraminic antidepressants, typical antipsychotics and antiemetics, immunosuppressants (cyclosporine, tacrolimus), anti-neoplastic agents - Withdrawal syndromes to benzodiazepines, ethanol, opiates	- Discontinuation of drug if possible - Propranolol otherwise
Akathisia	- Mainly antipsychotics, antiemetics, calcium-channel blockers and antidepressants (SSRIs > SNRIs) - Also benzodiazepines, carbamazepine, tetrabenazine, levodopa, lithium and sumatriptan	- Focal or generalized; reversible
Myoclonus	- Opiates, antidepressants, anticonvulsants, antipsychotics and antibiotics	
Chorea	- Antipsychotics and levodopa (peak-dose) - Anticonvulsants, anticholinergics, antihistaminic, antidepressants, lithium	- Generalized and hemichorea- Specific attention if diabetics
Dystonia/dyskinesia	- Dopamine blocking agents (antipsychotics, antiemetics) - SSRIs (if previous use of antipsychotics) - Levodopa	- Neuroleptic discontinuation or switch to clozapine/(deu) tetrabenazine/botulinum toxin/ GPi-DBS - Adapt levodopa dosage &/or add amantadine
Tics	- Dopamine blocking agents (antipsychotics, antiemetics) - Methylphenidate, anticonvulsants, antidepressants	- Vigilance in dehydrated or high dose of treatment or polypharmacy - Severity range from mild to severe
Drug-induced movement disorder emergencies (neuroleptic malignant syndrome, serotoninergic syndrome)	- Antipsychotics - Serotoninergic agents [antidepressants (SSRIs < SNRIs)] MAOIs (antidepressants, Parkinson's treatment, and antibiotics), triptans, buspirone, lithium)	
**Focus on specific drugs inducing movement disorders**		
**Drugs**	**Movement disorders**	**Comments**
Antipsychotics	Tardive syndromes (tardive dyskinesia, tardive dystonia, tardive stereotypies, tardive akathisia, tardive chorea, tardive myoclonus, tardive tremor, tardive tics and tourettism, but also tardive pain) Acute or subacute akathisia, dystonia Neuroleptic malignant syndrome.	- More frequent if 1^st^ generation - Antipsychotic discontinuation, switch to clozapine ± (deu)tetrabenazine ± botulinum toxin if focal ± Gpi-DBS if refractory
Antidepressants	Tremor, akathisia, myoclonus, chorea, tardive dyskinesia, tics, serotonin syndrome	
Lithium	Non-parkinsonian tremor, akathisia, chorea, myoclonus, cerebellar syndrome, and serotonin syndrome	- Low therapeutical index, plasma levels to be monitored - Frequent drug-drug interaction
Dopamine replacement therapy	Motor fluctuations and dyskinesia (peak dose and biphasic)	- Very frequent, context of PD
Anticonvulsants	Non-parkinsonian tremors, akathisia, myoclonus, tics, chorea	
Psychostimulants	Tics, stereotypies, tremor, myoclonus, chorea (“crack dancing”), orofacial and limb dyskinesia and dystonia, akathisia	

*DBS, deep brain stimulation; GPi, Globus pallidus interna; MAOIs, Monoamine Oxidase Inhibitors; PD, Parkinson's Disease; SNRIs, Serotonin and Norepinephrine Reuptake Inhibitors; SSRIs, Selective Serotonin Reuptake Inhibitors*.

### Neuroleptics, Dopamine Receptor Blocking Agents

A wide span of movement disorders can be induced by dopamine receptor blocking agents including antiemetics, calcium-channel blockers with additional D2 receptor blocking effects, and antipsychotics (117), with a prevalence that can vary depending on the type of drug, generation of antipsychotic, or duration of treatment (117, 118).

These movements can occur with various timings after the introduction of the drug. Tardive syndromes are characterized by repetitive rhythmic abnormal involuntary movements, caused by long-term exposure to dopamine receptor blockers. Drug-induced dystonia/dyskinesia represent around 4% of movement disorders in patients above the age of 50, raising to 6% of movement disorders in the elderly around 85 years old (115, 119). Children can also present with tardive syndromes induced by dopamine blocking agents, even though the prevalence is not easy to evaluate (120–122). Acute and subacute akathisia after introduction of a drug, and akathisia after withdrawal of a drug occur mostly with dopamine receptor blocking agents including antiemetics, calcium-channel blockers and antipsychotics, with a prevalence of 20–50% (117, 118). A recent meta-analysis estimated an incidence rate of akathisia of 7.7% with the newest approved antipsychotics, which could be underestimated due to underreporting (123).

Several different subtypes of tardive syndromes exist, based on the phenomenology: more frequently the classic tardive dyskinesia, tardive dystonia, tardive stereotypies, and more rarely tardive akathisia, tardive chorea, tardive myoclonus, tardive tremor, tardive tics and tourettism, but also tardive pain (117, 124). The most frequent ones, typically occurring in elderly women, are tardive dyskinesia, which typically comprise complex orofacial dyskinesias and stereotypies, or choreic neck and trunk movements. Tardive dystonia most typically occurs in young men and includes retrocollis, opisthotonic posturing, extension of the arms at the elbow, internal rotation at the shoulders and flexion of the wrists, and can be focal, segmental, or generalized (125–127). Tardive tourettism has been described as an uncommon form of tardive syndrome induced by the chronic use of dopamine blockers such as neuroleptics, not always meeting the diagnostic criteria for classic Tourette (124). Tics occur during treatment or following treatment discontinuation (128, 129). First-generation neuroleptics seem to favor vocal tics (in particular barking and coprolalia), compared to second-generation neuroleptics (128). Tics can sometimes be difficult to diagnose, and may be underreported as they are often intermingled with other abnormal movements (such as stereotypies) present in the original underlying neuropsychiatric disorder treated with dopamine blockers. Conversely, other drug-induced hyperkinetic movement disorders can also be misdiagnosed as tics (130).

While tardive syndromes typically occur during treatment or within 6 months of discontinuation with persistent symptoms lasting for at least 1 month, “withdrawal-emergent syndrome” lasts less than a month, then remits, and is more common in children (122, 131).

Movements can also have a subacute onset and be chronic reversible disorders: drug-induced Parkinsonism and acute or subacute akathisia. Whereas acute akathisia improves when removing the antipsychotics, tardive akathisia worsens during and after withdrawal.

Movements can also have an acute onset and be reversible disorders such as acute onset dystonia, which is seen mainly in young men and children, or neuroleptic malignant syndrome, which is of importance due to its potential severity.

Higher daily doses of neuroleptics, greater cumulative doses of neuroleptics, and greater severity of negative schizophrenic symptoms are predictive of severe tardive dyskinesia (132–134). There is a cumulative incidence of 60% of tardive dyskinesia and 23% of severe ones after 3 years of treatment. Prevalence of tardive movements seems lower with atypical (20%) than first generation (30%) antipsychotics, and the annual incidence is 6.5% with first generation vs. 2.6% with second generation neuroleptics (117, 127, 133, 135). Annual incidence rate of akathisia depends on the subtype of antipsychotic: 4% with quetiapine or olanzapine, 20% with other second-generation antipsychotics, and up to 50% with haloperidol.

The course of tardive syndrome is highly variable, from transient dyskinesia during the withdrawal period to irreversible dyskinesia despite withdrawal of the offending drug. When possible, discontinuation of the antipsychotic will help in some patients, knowing that a transient worsening can be observed at the time of discontinuation and that guidelines were proposed to avoid as much as possible such a phenomenon (122, 136–138). Of note, remission can occur as late as a few years after discontinuation of the causing drug (139). Switching to a drug with a lower affinity to D2 receptors, such as clozapine, is recommended in case pursuing neuroleptic treatment is mandatory (140). Antipsychotic reduction or switching are likely to have a positive clinical effect on reduction or reversibility of tardive dyskinesia, although not always statistically confirmed by some studies (141–144). Neither prophylaxis with anticholinergics nor symptomatic treatment with GABA agonists are recommended due to lack of evidence supporting their use (143, 145, 146). Treatments that can be added are dopamine depleting agents, VMAT2 inhibitors such as (deu) tetrabenazine or valbenazine in case of tardive syndromes, botulinum toxin for focal dystonia, and Gpi-DBS can be discussed in case of refractory disabling tardive dyskinesia (143, 147, 148). A seminal prospective study demonstrated a sustained 50% improvement in patients who had underwent GPi-DBS for tardive dystonia and dyskinesia (147). Similar or even better results were reported in a meta-analysis (149). DBS should, thus, always be considered in severe refractory tardive syndromes. The guidelines for the treatment of tardive syndromes are further detailed elsewhere (117, 127, 131, 145, 150). Treatment of tardive tics and tourettism include comprehensive behavioral intervention therapies, VMAT2 inhibitors such as tetrabenazine, topiramate and alpha agonists, botulinum toxin for focal disabling tics, and DBS for refractory cases (151, 152).

In case of hyperthermia or sudden onset increase of rigidity, neuroleptic malignant syndrome should be sought for, as it is a drug-induced life-threatening emergency. The general presentation usually comprises acute onset hyperthermia, rigidity, tremor, autonomic dysfunction, and mental status change. Early recognition of the appearance of these signs is important, to discontinue the treatments involved as early as possible and improve prognosis.

### Antidepressants

Antidepressants [Selective serotonin reuptake inhibitors (SSRIs) and imipraminic antidepressants] can cause tremor, akathisia, myoclonus, chorea, dyskinesia, tics, and obviously serotoninergic syndrome.

Dyskinesia under SSRIs mostly occur in case of previous neuroleptic use, and can resolve after SSRIs discontinuation (153–155). These dyskinesia triggered by antidepressants would be more frequent in diabetic patients and women, and if associated to anticholinergic treatments, but may be rare in patients never previously exposed to antipsychotic drugs (153, 156). Akathisia can be caused by all subclasses of antidepressants, with a prevalence of 5/1,000, maybe in particular fluoxetine. They are the main causative agents after dopamine blockers (116, 119). Antidepressant-induced myoclonus is usually reversible following withdrawal of the causing drug. Chorea can be antidepressant-induced even in a case of hemichorea without an underlying structural lesion. Antidepressants such as mirtazapine have also been reported as causative of tics (138).

Serotonin syndrome linked to serotoninergic agents among which SSRI but not only (157) is a life-threatening movement disorder emergency complication with a mortality rate ranging from 5 to 15%. Dehydration and high dosage of the medication involved are important predisposing factors. It presents with an acute onset of an association of hyperthermia, rigidity, myoclonus, autonomic dysfunction, and mental status change. Early recognition of its appearance is important to discontinue the treatments involved as early as possible and improve prognosis. It begins within hours to a day after initiating or increasing a serotonin-elevating drug and mostly happens when associating 2 or more drugs that increase serotonin, making polypharmacy a high risk situation (158). The main drugs involved are those with serotoninergic properties such as most of the antidepressants [i.e., SSRI, mono amine oxidase inhibitors (MAOI), imipraminic], but other causative drug should not be overlooked such as tramadol, dextromethorphan, antihistamines, triptans, buspirone, lithium, St John's wort, and their association. Neuropsychiatric symptoms range from nervousness and insomnia to impaired consciousness and hypomanic symptoms. Early neurological symptoms such as ataxia, akathisia, and mydriasis may precede myoclonus, ocular clonus, tremor, and hyperreflexia. Minor autonomic symptoms are tachycardia, dyspnea, diarrhea, and blood pressure dysregulation, whereas fever and diaphoresis are severe symptoms. Jitteriness, increase in anxiety, and irritability for 1–2 weeks after starting an antidepressant drug or increasing its dose can commonly occur without predicting the occurrence of a serotonin syndrome (159).

### Lithium

Lithium has a low therapeutic index. Symptoms of intoxication include delirium, fatigue, tremor, myoclonus, gait instability, and sometimes seizures (160). Focusing on abnormal hyperkinetic movements, Lithium intake can lead to non-parkinsonian tremor, akathisia, chorea, myoclonus, cerebellar syndrome, and serotonin syndrome. Tremor is a cause of lower observance and may be more frequent in older patients, particularly in older men and in case of intoxication, but not only (154, 161). Dehydration or co-prescription with diuretics, carbamazepine (CBZ) or tramadol are well-known circumstances leading to increasing these neurological adverse events. CBZ can be prescribed in the same patients already under Lithium, as it is also a mood stabilizer. The co-prescription can not only increase the risk of ataxia due to the sodium channel blocking properties of CBZ, but also increase the risk of hyperkinetic movements, as for instance myoclonus and dystonia, which are the most frequently reported with CBZ and its derivative oxcarbazepine (162).

### Anticonvulsants

Antiepileptics can cause non-parkinsonian tremor, notably sodium valproate, particularly in older women, but other antiepileptic drugs such as carbamazepine and phenytoin may as well. A predisposition to non-parkinsonian tremor induced by valproate, can be looked for with a personal and family history of tremor (e.g., Essential Tremor). Discontinuation or decrease of the causing drug can relieve the symptoms, but adding propranolol can be considered in case the drug needs to be maintained.

Carbamazepine can, also, occasionally be a cause of akathisia. Antiepileptics such as Lamotrigine, Carbamazepine, and Oxcarbazine have been reported to cause tics (129, 162–165).

Iatrogenic chorea can be caused by anticonvulsants, among which in particular phenytoin, gabapentin, carbamazepine, and valproate. Drug-induced chorea can present as generalized chorea, or hemichorea, even in the absence of an underlying structural lesion which could be suggested by the hemicorporeal location.

Anticonvulsants can also cause myoclonus. Almost all classes of drugs have been linked to myoclonus, from a focal to a generalized distribution, with or without being associated to encephalopathy (166).

### Dopamine Replacement Therapy

Dopamine replacement therapy can be associated with motor fluctuations and dyskinesia (peak dose and biphasic). In this context, a variety of movements can be observed from dystonia to akathisia, tremor, ballismus, and chorea, depending on the time of dosing. Levodopa-induced dystonia/dyskinesia are very frequent as they affect 90% of Parkinsonian patients after 9 years of treatment (131). They can also occur, even though less frequently, under treatment by dopamine agonists. Dopamine agonists can also occasionally lead to self-injurious behaviors, which can be quite disabling (132).

### Psychostimulants and Drugs of Abuse

Of note, several drugs of abuse and psychostimulants, such as cocaine, methylphenidate, amphetamine and metamphetamine, MDMA (Ecstasy), mephedrone, cathinone, and methcathinone, can cause hyperkinetic movements. These include mainly tics and stereotypies, tremor, myoclonus, chorea, orofacial and limb dyskinesia, and dystonia. Drugs of abuse such as cocaine can also occasionally cause akathisia and more frequently induce chorea-athetoid movements known as “crack dancing” (167–169), and also either induce or worsen underlying tics. Given the frequency of tics in the general population, a careful history of previous tics (as well as Obsessive Compulsive Disorder, Attention Deficit Hyperactive Disorder) and a family history should be sought as these may predispose to the development of tics with various drugs. Tics have been reported to be associated with psychostimulant use, in particular methylphenidate, which blocks the dopamine transporter (DAT), inhibiting the reuptake of dopamine and increasing dopamine in the synaptic cleft (129, 163, 170).

The pathophysiological common underlying mechanism is the effect of those psychostimulant drugs on the monoaminergic systems (dopamine, Norepinephrine, serotonin). The cornerstone of treatment is the cessation of drug intake.

In case of tremor in the context of chronic intoxication, benzodiazepines, ethanol, and opiates withdrawal syndromes should also be kept in mind. Opiates are also a main cause of iatrogenic myoclonus.

The reader can read further details elsewhere (170).

## Genetic Diseases

Genetic diseases are rare causes of treatable hyperkinetic movement disorders (summarized in Table 5). Onset in childhood or adolescence, presence of a family history and a chronic course of the disease are clues to a genetic cause, although adult onset, sporadic cases and acute presentations may be seen. Disorders that may be considerably improved by deep brain stimulation, such as dystonia due to *TOR1A, KMT2B*, or *SGCE* mutations, have not been included in this review.

**Table 5:** Genetic causes of treatable hyperkinetic movement disorders.

**Cause**	**Movement disorders**	**Diagnostic clues**	**Laboratory findings**	**Treatment**
Paroxysmal kinesigenic dyskinesia	Isolated paroxysmal kinesigenic dyskinesia		*PRRT2* mutation	Antiepileptics (Na channel blockers)
ADCY5-related dyskinesia	Pleiotropic paroxysmal dyskinesia, orofacial myoclonus	Nocturnal episodes, hypotonia, fluctuations	*ADCY5* mutation (possibly mosaïc)	Caffeine
GLUT1 deficiency	paroxysmal exercise-induced dyskinesia	Seizures, episodic ataxia, intellectual deficiency, worsening with fasting or exercise	Low CSF glucose levels, *SLC2A1* mutation	Ketogenic diet Triheptanoin
Dopa-responsive dystonia	Dystonia, paroxysmal exercise-induced dyskinesia	Motor fluctuations, sleep benefit, oculogyric crises	Abnormal CSF neurotransmitters, *GCH1, TH, SPR* or *PTPS* mutation	Levodopa
Pyruvate dehydrogenase deficiency	Dystonia, paroxysmal dystonia	Episodic ataxia Bipallidal lesions on MRI	High lactate and pyruvate levels, *PDHA1, PDHA2, PDHX* or *DLAT* mutations	Thiamine Ketogenic diet
Glutaric aciduria type 1	Dystonia	Acute encephalopathy episodes	Elevated glutaric acid in plasma and/or urine, *GCDH* mutations	Low-lysine diet, carnitine supplementation
Cerebral folate transport deficiency	Myoclonus, chorea	Developmental delay, seizures, ataxia	Low 5-MTHF in the CSF, *FOLR1* mutations	Folinic acid
Cerebrotendinous xanthomatosis	Myoclonic dystonia	Chronic diarrhea, early cataract, ataxia	High plasma cholestanol, *CYP27A1* mutations	Chenodoxycholic acid
Wilson's disease	Tremor, dystonia, parkinsonism, chorea	Striatum, thalamus, brainstem, cerebellum T2 hyperintensities	Low serum ceruleoplasmin and copper, high urinary copper, *ATP7B* mutations	Copper chelating agents Zinc
Niemann Pick disease type C	Dystonia, chorea, myoclonus, parkinsonism,	Ataxia, supranuclear gaze palsy	Elevated serum oxysterols, *NPC1* or *NPC2* mutations	Miglustat
Ataxia with vitamin E deficiency	Dystonia, head tremor	Ataxia	Very low plasma vitamin E, *TTPA* mutations	High doses of Vitamin E
Biotin-thiamin responsive basal ganglia disease	Dystonia	Episodes of encephalopathy, ataxia Caudate nuclei and putamen T2 hyperintensities	*SLC19A3* mutations	High doses of biotin and thiamine
Dystonia/parkinsonism, hypermanganesemia, polycythemia and chronic liver disease	Dystonia, parkinsonism	Basal ganglia T1 hyperintensities	Elevated blood manganese, *SLC30A10* mutations	Manganese chelation therapy

*Na, sodium; CSF, cerebrospinal fluid*.

### Paroxysmal Movement Disorders

#### Paroxysmal Kinesigenic Dyskinesia

Paroxysmal kinesigenic dyskinesia is an autosomal-dominant disorder caused in most cases by a mutation in the *PRRT2* gene, coding for a synaptic protein (171, 172). Prevalence is estimated to be of 1 in 150,000. It is characterized by attacks of dystonia and/or chorea triggered by sudden voluntary movement that last less than a minute but may happen up to 100 times a day. Onset is usually in childhood or adolescence, with usual improvement in early adulthood, so that onset after the age of 20 years is a red flag for a secondary cause. Association with benign infantile epilepsy in the patient and/or family members is quite frequent. Early diagnosis is important, as treatment with low doses of antiepileptic drugs, especially sodium channel blockers (carbamazepine, lamotrigine, zonisamide) can suppress or dramatically reduce attacks (173, 174).

#### Paroxysmal Exercise-Induced Dyskinesia

Paroxysmal exercise-induced dyskinesia is characterized by attacks of dystonia and/or chorea triggered by prolonged exercise, which last for several minutes (171). Mutations in *SLC2A1* are the main causes of PED, detailed in the next paragraph (GLUT1 deficiency). Other treatable causes of PED include Dopa-responsive dystonia and Pyruvate dehydrogenase deficiency (see next paragraph), as well as Parkinson's disease (175).

#### ADCY5-Related Dyskinesia

Heterozygous mutations in *ADCY5*, coding for adenylate cyclase type 5, a protein involved in dopamine signaling in the striatum, cause a rare childhood-onset mixed hyperkinetic movement disorder. The presence of axial hypotonia, orofacial myoclonus, and various types of paroxysmal movement disorders (in particular nocturnal episodes) as well as marked fluctuations of the motor state are strong clues to the diagnosis (176). Treatment with caffeine can induce dramatic improvement of paroxysmal episodes, and should therefore be tried in all patients (177, 178).

### Neurometabolic Diseases

Most genetic disorders presenting with hyperkinetic movement disorders in childhood or adulthood are neurometabolic diseases (NMD), which are characterized by enzymatic deficiencies or defects in proteins involved in cellular metabolism. Movement disorders are a frequent, and sometimes the only presenting manifestation of NMD and may be present throughout the course of the disease. In addition, a given NMD can cause a variety of movement disorders, while a given movement disorder can occur in a variety of NMD. Complex movement disorders, marked orofacial involvement, associated symptoms, and brain MRI abnormalities should evoke the possibility of an underlying neurometabolic disorder. The phenomenology of movement disorders in NMD is partly age dependent: young patients tend to have an axial hypotonia/hyperkinetic limb pattern, whereas fixed dystonia and Parkinsonism predominate in older patients (179).

#### GLUT1 Deficiency

GLUT1 deficiency is caused by mutations in *SLC2A1*, which codes for a glucose transporter, resulting in a cerebral energy deficit. The phenotypic spectrum is wide, ranging from severe encephalopathy manifesting in infancy to isolated paroxysmal exercise-induced dyskinesia occurring later in life (180, 181). Manifestations can include intellectual deficiency, microcephaly, dystonia, chorea, ataxia, spasticity, and paroxysmal episodes, including seizures and paroxysmal dyskinesia. Worsening with fasting or exercise is a good diagnostic clue. Diagnosis is based on low cerebrospinal fluid (CSF) glucose levels in a patient with clinical manifestations consistent with GLUT1 deficiency, whereas low CSF lactate levels is a good supportive feature. Genetic analysis can support the diagnosis (182). Standard treatment consists of a ketogenic diet, with the goal of providing alternative energy substrates to the brain. It is usually very effective. Alternative treatments are however needed, as the diet is hard to follow and is ineffective in rare patients. Triheptanoin may be an option, having shown dramatic and sustained improvement on paroxysmal dyskinesia in an open-label trial (183–185).

#### Dopa-Responsive Dystonia

Dopa-responsive dystonia is mostly caused by heterozygous mutations in the GTP-cyclohydroxylase-1 (*GCH1*) gene. It can rarely be caused by autosomal recessive mutations in the tyrosine hydroxylase (TH), sepiapterine reductase (SPR), or pyruvoyl tetrahydropterin synthase (PTPS) genes (186). GTP-cyclohydroxylase 1 catalyzes the first step in the synthesis of tetrahydrobiopterin, which is essential for dopamine synthesis. Dopa-responsive dystonia (DRD) usually manifests as childhood-onset dystonia with motor fluctuations, but atypical forms are not uncommon. They include adult onset generalized dystonia, focal dystonia, paroxysmal exercise-induced dyskinesia, task-specific dystonia, myoclonus dystonia, isolated Parkinsonism, and spastic paraparesis (187). Postural tremor, axial hypotonia, and oculogyric crises can be observed, the latter suggesting a cause other than a *GCH1* mutation (188). Diagnosis is crucial, as there is a dramatic response to treatment by low-dose levodopa. A levodopa trial and/or a CSF neurotransmitters analysis or direct genetic testing if easily available should therefore be proposed in all patients with early-onset dystonia (<30 years of age) or paroxysmal exercise-induced dyskinesia. Of note, another neurotransmitter disease, aromatic L-amino acid decarboxylase (AADC) deficiency, which causes developmental delay and movement disorders, may be effectively treated by gene therapy (189).

#### Pyruvate Dehydrogenase Deficiency

Pyruvate dehydrogenase deficiency is caused by mutations in genes coding for the mitochondrial pyruvate dehydrogenase complex that has a crucial role in cellular metabolism (*PDHA1* in most cases, more rarely *PDHA2, PDHX*, or *DLAT*) (190–192). Most patients present with severe early-onset encephalopathy, whereas others may present with prominent movement disorders, mainly static and/or paroxysmal dystonia or episodic ataxia. In rare cases, the phenotype may be as mild as isolated PED (193). Investigations typically reveal high lactate and pyruvate levels, a low lactate to pyruvate ratio in blood and CSF and bipallidal lesions on brain MRI, but may be normal in milder cases. Treatment with high doses of thiamine, a co-factor of the pyruvate dehydrogenase complex, can be effective in some patients, whereas others respond better to a ketogenic diet (193, 194).

#### Glutaric Aciduria Type 1

Glutaric aciduria type 1 is an autosomal recessive disorder caused by mutations in the *GCDH* gene leading to Glutaryl-coenzyme A dehydrogenase deficiency (195, 196). Acute encephalopathy episodes in early childhood are accompanied by irreversible lesions of the basal ganglia, so that early and sudden onset of movement disorders are common in this disorder (179). Younger patients usually present with axial hypotonia and mobile dystonia that give way to fixed generalized dystonia as they age. Diagnosis is either made on routine newborn screening, now available in many countries, or with plasma and urine organic acids chromatography. Early diagnosis and treatment (with low-lysine diet and carnitine supplementation) and intensified emergency treatment during periods of intercurrent illnesses considerably improve the prognosis (197).

#### Cerebral Folate Transport Deficiency

Primary cerebral folate deficiency is an autosomal recessive disorder caused by mutations in *FOLR1*, a gene coding for a folate receptor. The onset is usually in early childhood, combining developmental delay, seizures, ataxia, myoclonus, and chorea (196, 198). The brain MRI shows hypomyelination and cerebellar atrophy. Diagnosis is suggested by very low levels of 5-methyltetrahydrofolate in the CSF and confirmed by molecular analysis. Early diagnosis and treatment with folinic acid significantly improves symptoms, allowing for complete recovery in some cases (198, 199).

#### Cerebrotendinous Xanthomatosis

Cerebrotendinous xanthomatosis is an autosomal recessive lipid storage disease caused by mutations in *CYP27A1* resulting in an accumulation of cholestanol. Most symptoms start in childhood or adolescence, but the disorder is usually diagnosed in adulthood (200). Chronic diarrhea is often the first manifestation of the disease in infancy, followed by bilateral cataract between 5 and 15 years of age. Tendon xanthomas, particularly of the Achilles tendon, are good clues to the diagnosis, but may be absent. The neurological manifestations of the disease occur later, usually during adolescence or early adulthood. Among motor symptoms, cerebellar and pyramidal features are the most frequent. In addition, peripheral neuropathy is common in adults with cerebrotendinous xanthomatosis (CTX). Cognitive impairment, epilepsy and psychiatric disturbances can also be part of the neurological manifestations. CTX-related movement disorders comprise dystonia, myoclonus, tremor and Parkinsonism, and may occasionally be the presenting symptoms (201). The brain MRI typically shows T2-weighted hyperintensities of the dentate nuclei. Diagnosis is suggested by high plasma cholestanol levels and confirmed by molecular analysis. While treatment with chenodeoxycholic acid can lower cholestanol levels and prevent progression, the effect on existing symptoms is variable (202). Measurement of plasma cholestanol should thus be pursued in any child with infantile chronic diarrhea or cataract, and in adolescents or young adults with unexplained cerebellar ataxia, pyramidal signs, peripheral neuropathy and/or hyperkinetic movements.

#### Wilson's Disease

Wilson's disease is an autosomal recessive disorder due to mutations in *ATP7B*, coding for a copper-transporting protein. Prevalence is estimated to be of 1 in 30,000. It can present as liver disease, most commonly in children, or as a neuropsychiatric disorder, more frequently in adults. Age of onset ranges from 2 to 72 years (203). Wilson's disease should be suspected in patients presenting with tremor, dystonia, dysarthria and/or akineto-rigid syndrome, with behavioral changes or cognitive deterioration.

There are three main types of neurologic manifestations: (i) a dystonic form, characterized by major orofacial involvement, leading to *risus sardonicus*, swallowing difficulties and dysarthria; (ii) a parkinsonian form, characterized mainly by akinesia and rigidity; (iii) a tremoric form, with a progressive slow and high amplitude limb-onset postural and action tremor that may spread to the whole body. The various movement disorders (tremor, dystonia, and Parkinsonism) can be combined, and chorea is rarely present. Dystonic forms are mostly seen in children and young adults, whereas the tremoric and akineto-rigid forms are more common in older individuals. The diagnosis is made on the combination of low serum ceruloplasmin and copper concentrations, high urinary copper levels, suggestive brain MRI abnormalities, and Kayser-Fleisher rings (copper deposits in the periphery of the cornea) on slit lamp examination. The brain MRI usually shows diffuse and symmetrical T2-weighted hyperintensities in the striatum, thalamus, brainstem, cerebellum, and possibly the white matter. The relative exchangeable copper (REC) level (exchangeable copper/total serum copper) is the most sensitive and specific diagnostic tool (204). Confirmation is made by molecular analysis of the *ATP7B* gene. Treatment consists of a low copper diet along with copper chelating agents such as D-penicillamine and Trientine or Zinc, which interferes with the absorption of copper. Symptoms may worsen at the treatment onset, with improvement occurring only after 3–6 months of treatment. Liver transplantation could be considered as a rescue option in patients with severe neurological forms (albeit without severe necrotic lesions) that are resistant to anticopper therapies (205).

#### Niemann Pick Disease Type C

Niemann Pick disease type C (NPC) is an autosomal recessive lysosomal disorder due to mutations in *NPC1* or *NPC2* that code for intracellular cholesterol transporters. Onset can occur at any age, but is more common before the age of 30. Movement disorders are seen in 50–60% of the patients, manifesting mostly as generalized dystonia, but sometimes involving Parkinsonian signs, chorea, or myoclonus (206). Main diagnostic clues are vertical supranuclear gaze palsy, cerebellar ataxia, behavioral changes, cognitive deterioration, and enlarged liver or spleen (207). Dysarthria and swallowing difficulties are frequent. The brain MRI can show non-specific atrophy of the cortex, cerebellum, and/or brainstem. Diagnosis is evoked by a positive filipin test on skin fibroblasts or elevated serum levels of oxysterols, and confirmed by molecular analysis. Treatment with oral miglustat can stabilize key neurological manifestations of NPC, emphasizing the need for early diagnosis (208).

#### Ataxia With Vitamin E Deficiency

Ataxia with Vitamin E deficiency is an autosomal recessive disorder caused by mutations in *TTPA*, coding for the alpha-tocopherol transfer protein. Age of onset varies from 2 to 37 years (209). Although ataxia with Vitamin E deficiency (AVED) manifests mostly as a Friedreich-like ataxia, it can also present as dystonia (210). Head tremor, in particular, is a frequent manifestation of the disease. Diagnosis is suggested by very low plasma vitamin E concentrations with a normal lipoprotein profile and confirmed by molecular analysis. The treatment consists of lifelong high doses (800–1,500 mg/day) oral vitamin E supplementation. Plasma vitamin E should be monitored regularly and maintained in the high normal range (209). Early diagnosis and treatment are crucial, as some symptoms (ataxia and intellectual deterioration) can be reversed. Evaluation of relatives at risk is important, as vitamin E supplementation in presymptomatic individuals can prevent manifestations of the disease.

#### Biotin-Thiamine-Responsive Basal Ganglia Disease and Other Treatable Mitochondrial Disorders

Biotin-thiamine-responsive basal ganglia disease is an autosomal recessive disorder caused by mutations in the *SLC19A3* gene, coding for a thiamine transporter. Onset is usually in childhood or adolescence, but adult onsets have also been reported (211, 212). It is characterized by recurrent episodes of subacute encephalopathy with confusion, seizures, ataxia, dystonia, external ophtalmoplegia, and dysphagia. If left untreated, patients develop permanent generalized dystonia. The brain MRI shows characteristic bilateral T2-weighted hyperintensities in the caudate nuclei and putamen, which can evolve to necrosis in the later stages of the disease. Early diagnosis is crucial, as administration of high doses of biotin (5–10 mg/kg/day) and thiamine (300–900 mg/day) results in dramatic improvement within days.

Thiamine pyrophosphokinase deficiency, caused by biallelic *TPK1* mutations, is also characterized by episodes of encephalopathy, ataxia, and dystonia (213). Treatment involves high-dose thiamine supplementation, with variable outcomes (214).

Coenzyme Q10 deficiency due to biallelic mutations in the *ADCK3* gene, also known as ARCA2 (autosomal recessive cerebellar ataxia 2), may also present with movement disorders (myclonus, dystonia, tremor) in addition to ataxia (215). Ubiquinone supplementation therapy (10–15 mg/kg/day) has been shown to improve movement disorders in some patients (216).

#### Dystonia/Parkinsonism, Hypermanganesemia, Polycythemia and Chronic Liver Disease

The disorder of dystonia/Parkinsonism, hypermanganesemia, polycythemia, and chronic liver disease is an autosomal recessive disorder caused by mutations in *SLC30A10*, coding for a manganese transporter (217). The onset can be in childhood, manifesting mainly as generalized dystonia, or in adulthood, typically manifesting as dopa-resistant Parkinsonism. The brain MRI shows bilateral and symmetric T1-weighted hyperintensities of the globus pallidus, putamen, caudate, and dentate nuclei. Laboratory findings usually include elevated blood manganese concentrations, polycythemia, and elevated transaminases. However, chronic liver disease or hypermanganesemia may be absent early on (218). Diagnosis is confirmed by molecular analysis. Chelation therapy with intravenous disodium calcium edetate and iron supplementation can normalize manganese levels, improve neurologic symptoms and stop liver disease. Of note, mutations of *SLC39A14* are another cause of dystonia/Parkinsonism and hypermanganesemia, without polycythemia and liver disease. Treatment also involves chelation therapy (219).

## Functional Hyperkinetic Movement Disorders

Functional movement disorders account for 2–4% of all movement disorders and may majorly improve or even disappear with rapid and adequate management. More details on this frequent and treatable condition can be found elsewhere (220, 221).

## Author Contributions

AM and ER designed the article. AM, BG, SF, AL, L-LM, and ER drafted and revised the paper. All authors contributed to the article and approved the submitted version.

## Conflict of Interest

The authors declare that the research was conducted in the absence of any commercial or financial relationships that could be construed as a potential conflict of interest.

## Publisher's Note

All claims expressed in this article are solely those of the authors and do not necessarily represent those of their affiliated organizations, or those of the publisher, the editors and the reviewers. Any product that may be evaluated in this article, or claim that may be made by its manufacturer, is not guaranteed or endorsed by the publisher.
